# A phase I trial of ANG1/2-Tie2 inhibitor trebaninib (AMG386) and temsirolimus in advanced solid tumors (PJC008/NCI♯9041)

**DOI:** 10.1007/s10637-015-0313-8

**Published:** 2015-12-19

**Authors:** Joanne W. Chiu, Sebastien J. Hotte, Christian K. Kollmannsberger, Daniel J. Renouf, David W. Cescon, David Hedley, Sue Chow, Jeffrey Moscow, Zhuo Chen, Meghan Perry, Ivan Diaz-Padilla, David Tan, Hal Hirte, Elaine McWhirter, Helen Chen, Lillian L. Siu, Philippe L. Bedard

**Affiliations:** Division of Medical Oncology & Hematology, Princess Margaret Cancer Centre, University Health Network, Suite 5-125, 610 University Avenue, Toronto, ON M5G 2M9 Canada; Department of Medicine, University of Toronto, Toronto, ON Canada; Juravinski Cancer Centre and Escarpment Cancer Research Institute, Hamilton, ON Canada; British Columbia Cancer Agency, Vancouver, BC Canada; National Cancer Institute, Cancer Therapy Evaluation Program, Rockville, MD USA

**Keywords:** AMG386, Trebananib, Temsirolimus, Phase 1, Combination therapy

## Abstract

*Background* There is crosstalk between the ANG-Tie2 and the PI3K/Akt/mTOR pathways. Combined ANG1/2 and mTOR blockade may have additive anti-cancer activity. The combination of trebananib, an inhibitor of ANG1/2-Tie2 interaction, with temsirolimus was evaluated in patients with advanced solid tumors to determine tolerability, maximum tolerated dose (MTD), and preliminary antitumor activity. *Methods* Patients were enrolled using 3 + 3 design, and were given intravenous trebananib and temsirolimus on Day 1, 8, 15 and 22 of a 28-day cycle. Dose limiting toxicities (DLTs) were evaluated during cycle 1. Peripheral blood was collected for evaluation of Tie2-expressing monocytes (TEMs) and thymidine phosphorylase (TP). Sparse pharmacokinetic (PK) sampling for trebananib drug levels was performed on Day 1 and 8 of cycle 2. *Results* Twenty-one patients were enrolled, 6 at dose level (DL) 1, 7 at DL −1, and 8 at DL −2. No effect of temsirolimus on trebananib PK was observed. The most common treatment-related adverse events (AEs) were: fatigue (81 %), edema (62 %), anorexia (57 %), nausea (52 %), rash (43 %) and mucositis (43 %). The most common grade ≥ 3 AEs included lymphopenia (28 %) and fatigue (28 %). The MTD was exceeded at DL-2. Of 18 response evaluable patients, 1 partial response was observed (ER+/HER2−/*PIK3CA* mutant breast cancer) and 4 patients had prolonged SD ≥ 24 weeks. No correlation with clinical benefit was observed with change in number TEMs or TP expression in TEMs with treatment. *Conclusions* The MTD was exceeded at trebananib 10 mg/kg weekly and temsirolimus 20 mg weekly, with frequent overlapping toxicities including fatigue, edema, and anorexia.

**Electronic supplementary material:**

The online version of this article (doi:10.1007/s10637-015-0313-8) contains supplementary material, which is available to authorized users.

## Introduction

Approved agents against the vascular endothelial growth factor (VEGF) pathway and its receptor (VEGFR), such as bevacizumab, aflibercept, and sunitinib, provide proof of principle of targeting angiogenesis. However, the efficacy of these anti-VEGF therapies is suboptimal and the duration of response is often short-lived. It has been postulated cancer may not respond to anti-VEGF therapy or regain growth potential through the development of evasive resistance. Among the proposed mechanisms include up-regulation of alternative pro-angiogenic signals, protection of tumor vasculature by recruitment of pro-angiogenic inflammatory cells or by increasing protective pericyte coverage [1].

Inhibition of the angiopoietin-Tie2 system presents an attractive strategy to circumvent resistance to anti-VEGF therapy. Angiopoietins (ANG) are a family of secreted ligands of the endothelial receptor Tie2 that play a critical role in initiation of tumor angiogenesis, tumor inflammation and metastasis [2]. There are 3 members of ANG known to participate in angiogenesis in human. ANG-1 has both pro- and antiangiogenic functions, but the exact role has not been well characterized [3–6]. ANG-2 is believed to be the primary ANG involved in angiogenesis in malignant setting. It is frequently up-regulated at areas of angiogenesis within tumors. Elevated expression of this ligand is associated with more advanced disease and poor prognosis [7–10]. The function of ANG-4 is unclear, as it has a restricted pattern of expression in lung [11].

Trebananib (AMG386) is a first-in-class selective antagonist peptide-Fc fusion protein inhibiting the interaction between ANG-1, ANG-2 and Tie2. In xenograft models, this agent demonstrated potent anti-tumor activity, accompanied by suppressed endothelial cell proliferation [7]. The phase 1 clinical trial of trebananib in patients with advanced solid tumors showed favorable tolerability and preliminary anti-tumor activity [12]. The most common toxicities were fatigue and peripheral edema; few patients experienced treatment-related toxicities greater than grade 1. The maximum-tolerated dose (MTD) was not reached. One patient with refractory ovarian cancer achieved partial response associated with prolonged treatment of 156 weeks. Trebananib given at 10 mg/kg intravenously (IV) weekly can be safely combined with several standard chemotherapy regimens [13].

In view of its favorable safety profile, it was hypothesized that trebananib may be suitable for combination therapy with other anti-angiogenic agents. The mammalian target of rapamycin (mTOR) is a protein kinase of the PI3K/Akt signaling pathway with known critical role in cancer growth and tumor angiogenesis [14]. There is crosstalk between the ANG-Tie2 and the PI3K/Akt/mTOR pathways, as activation of Tie2 receptor leads to recruitment of the p85 regulatory subunit of PI3K and downstream activation of Akt [15]. ANG-1 modulates the survival, migration and sprouting of endothelial cells through PI3K/Akt activation. Temsirolimus is a mTORC1 inhibitor approved for the treatment of advanced renal cell carcinoma (RCC) and mantle-cell lymphoma [16, 17]. Here, we report the results of a phase 1b trial of trebananib in combination with temsirolimus in patients with advanced solid tumors.

## Patients and methods

### Study objectives

The primary objectives of this phase 1b trial were to evaluate the safety and tolerability of escalating doses of trebananib in combination with temsirolimus, to explore the dose-limiting toxicities (DLTs), and to determine the recommended phase 2 dose (RP2D). The secondary objectives were to evaluate preliminary tumor response in patients with advanced solid tumors and to assess the pharmacodynamics effects with the goal of identifying potential predictive markers that warrant further exploration.

### Eligibility criteria

Inclusion criteria included age of 18 years or older with histologically or cytologically confirmed advanced solid tumors, and for which standard anticancer treatment did not exist or were no longer effective; Eastern Cooperative Oncology Group (ECOG) performance status of 0–1; life expectancy ≥12 weeks; Response Evaluation Criteria in Solid Tumors (RECIST) v1.1 measurable disease; well-controlled blood pressure; adequate bone marrow, renal, liver functions and lipid profile. Key exclusion criteria included central nervous system metastasis; coagulopathy or history of clinically significant bleeding within 6 months; venous or arterial thromboembolism within 12 months; unresolved toxicities from prior systemic therapy ≥ grade 2 by CTCAE version 4.0; prior treatment by trebananib or medication targeting ANG or Tie2 receptor; clinically significant cardiovascular disease within 12 months; major surgery within 28 days; treatment within strong immune modulators within 30 days; non-healing wound, ulcer, or fracture; chemotherapy or radiotherapy within 4 weeks or other investigational drug within 21 days; concomitant administration of strong CYP3A4 inhibitors or inducers. Written informed consent was obtained from all participants included in the study prior to screening procedures. This study was approved by local Institutional Review Boards.

### Study design and dose de-escalation

Dose exploration to identify the MTD followed a 3 + 3 design. Trebananib was administered intravenously (IV) over 60 min, followed by temsirolimus IV over 30–60 min on day 1, 8, 15, and 22 of each 28-day cycle. The starting dose level (DL1) of trebananib 15 mg/kg and temsirolimus at its approved dose of 25 mg was based on the expected tolerability of the combination. Due to the toxicities encountered, reduced dose levels of trebananib 15 mg/kg and temsirolimus 20 mg weekly (DL-1) and trebananib 10 mg/kg and temsirolimus 20 mg weekly (DL-2) were explored.

### Definition of DLTs and MTD

Toxicities were graded according to the National Cancer Institute Common Terminology Criteria for Adverse Events (NCI CTCAE) v4.0. DLTs were determined by treatment-related toxicities that occurred during cycle 1. DLT criteria included non-hematologic toxicity that was grade (G) 4, or G3 which did not resolve to G1 within 48 h; toxicities that resulted in failure to receive ≥75 % of the pre-planned doses of trebananib and/or temsirolimus; neutropenia <0.5 × 10^9^/L lasting ≥7 days, neutropenic fever, thrombocytopenia <25 × 10^9^/L or thrombocytopenic bleeding. The MTD was defined as the highest DL at which ≤1/6 patients experienced DLTs during first cycle.

### Evaluation of safety

Toxicities were recorded for patients who received one or more doses of trebananib and temsirolimus. Vital signs and weight were measured weekly before each dose of study treatment during cycle 1 and then pre-dose before every subsequent cycle. Complete blood count was assessed before every treatment. Serum chemistry including cholesterol and triglycerides were assessed pre-dose weekly during cycle 1 and the pre-dose day 1 and day 15 of subsequent cycles. Urine protein was measured via dipstick pre-dose day 1 and day 15 of cycle 1 and then pre-dose day 1 of every subsequent cycle. Serum for assessment of anti-trebananib antibodies was collected pre-dose Cycle 1, Day 1, pre-dose Cycle 2, Day 8, and at the end of treatment (EOT) visit approximately 30 days after the last dose of trebananib.

### Evaluation of tumor response

Patients with measurable disease were evaluated for response by imaging or clinical examination every 8 weeks using the Response Evaluation Criteria in Solid Tumors (RECIST) version 1.1 [18]. A confirmatory scan was obtained 4 weeks following initial documentation of an objective response.

### Sparse pharmacokinetics (PKs) and anti-trebananib antibodies

Plasma samples were collected at cycle 2 day (D) 1 (pre-dose and end of infusion at 10 min) and cycle 2 D8 (pre-dose) for trebananib concentrations, and at cycle 1 D1 (pre-dose), cycle 2 D8 (pre-dose) and EOT for presence of anti-trebananib antibodies. Both analyses of PKs anti-trebananib antibodies were performed by Amgen using previously published assays [12].

### Correlative studies

Peripheral blood was collected for correlative studies on cycle 1 D1, D3, D8 prior to drug administration (except D3), on cycle 2 D1 prior to and 4 h after drug administration, and at the EOT visit. Tie2 is expressed by monocytes and the role of Tie-2 expressing monocytes (TEMs) as a potential marker of response was explored. Thymidine phosphorylase (TP) is an angiogenic enzyme, which is increased in TEM upon Tie2 stimulation. Two separate staining procedures were employed: extracellular Tie2 staining with phycoerythrin-conjugated anti-Tie2 antibody after hypotonic red blood cell lysis; intracellular TP staining in a second aliquot of blood followed fixation with formaldehyde and permeabilization with Triton X-100, using indirect TP staining. CD45 and CD33 were used for gating on monocytes and lymphocytes. To normalize results, a ratio of the Median Fluorescence Intensities (MFI) between Monocytes and Lymphocytes (M/L ratio) was calculated for each sample. Associations between baseline Tie2 and TP M/L ratios, and changes in M/L ratios over time with best radiographic response in target lesions were explored. The assay has been verified with blood samples from healthy volunteers [19].

Circulating angiogenic factors (CAFs) including, soluble vascular cell adhesion molecule 1 (sVCAM-1), placental growth factor (PlGF), and VEGF-A were measured at the aforementioned time points using enzyme-linked immunosorbent assay (ELISA) kits from R&D System (Minneapolis, MN, USA). The assays were performed according to manufacturer’s instructions. We hypothesized that these pharmacodynamic markers would rise following the administration of studied drugs. For *PIK3CA* mutation status, formalin fixed paraffin embedded (FFPE) archival tumor tissue was collected. Molecular characterization was performed using the customized multiplex MassARRAY Sequenom (Sequenom, San Diego, U.S.A.) PMH v1.0 panel or Illumina MiSeq TruSeq Amplicon Cancer Panel (Illumina, San Diego, U.S.A.) in the University Health Network CLIA-certified laboratory. These results were correlated with tumor response.

### Statistical analysis

Descriptive statistics were used to summarize demographic, safety, antitumor activity, and correlative results. Comparison between treatment groups was performed using t-test. Continuous data were presented as mean and standard error; and categorical data were summarized using frequency and percentage. Difference with *p*-value of <0.05 is considered statistically significant.

## Results

### Patient characteristics

From April 2012 to March 2014 a total of 21 patients were enrolled. Patient characteristics are summarized in Table 1. The reasons for discontinuation included progressive disease (*n* = 11), toxicity (*n* = 5), withdrawal of consent (*n* = 3), death on study during cycle 1 (*n* = 1), and development of brain metastasis during cycle 1 (*n* = 1). All 3 patients who withdrew consent were due to intolerable toxicity, including one patient who remained on study for 37 weeks with best response of stable disease (SD) before withdrawal.

**Table 1:** Patient characteristics

	Characteristics	No. %
Gender	Female Male	16 (76 %) 5 (24 %)
Age	Median Range	58 32–74
ECOG performance score	0 1	5 (24 %) 16 (76 %)
Primary tumor type	Ovary Colon Lung Neuroendocrine Others^a^	4 4 2 2 9
Prior therapy	Chemotherapy	21 (100 %)
Previous chemotherapy regimens	1 2 3 ≥4 Median (range)	2 (9 %) 2 (9 %) 8 (38 %) 9 (43 %) 3 (1–11)

^a^Others: include one of each of breast cancer, gastric cancer, gallbladder cancer, thyroid cancer, bladder cancer, renal cell carcinoma, endometrial mixed carcinoma, bone chordoma, and endometrial sarcoma

### DLT and MTD

A summary of DLT events and the dose levels explored are presented in Table 2. In DL 1, 1 of 6 patients experienced DLT with G2 pneumonitis. Due to frequent G1 and 2 AEs including fatigue, nausea, edema, thrombocytopenia, mucositis and rash (Table 3) in DL1, de-escalation to DL-1 was explored. DLTs were experienced by 2 patients, including G3 mucositis and intolerable G2 limb edema leading to a treatment interruption that did not allow for initiating cycle 2 until more than 14 days after the planned start date. Further de-escalation to DL −2 was explored, and 2 of 6 evaluable patients experienced DLTs, including one episode of G4 hypertriglyceridemia and one death. This patient who died had advanced sigmoid colon cancer and failed 4 lines of systemic therapy before joining the study. On cycle 1 day 7 he presented with persistent diarrhea and was admitted. He then developed fever related to *Clostridium Septicum* bacteremia. Despite aggressive treatment, he rapidly deteriorated and succumbed to multi-organ failure. A post-mortem examination showed that the patient had a transmural recurrence at the site of a prior sigmoid colon anastomosis, with a walled off perforation at this site with necrosis and abscess formation. These AEs were considered to be probably related to study treatment. MTD was exceeded at DL-2 and no further dose de-escalation was explored.

**Table 2:** Dose escalation results

Dose level	Trebananib (mg/kg)	Temsirolimus (mg)	No. patients treated (*n* = 21)	No. DLT	DLT
1	15	25	6 (1 IE)	1	G2 pneumonitis
−1	15	20	7	2	G3 mucositis G2 intolerable edema
−2	10	20	8 (2 IE)	2	G4 sepsis, G4 diarrhea, G5 multi-organ failure (same patient) G4 hypertriglyceridemia

*IE* inevaluable

**Table 3:** Frequent or significant adverse events (possibly related to treatment)

Adverse events	All grades No. Patient (%)	Dose level (DL1/-1/-2)	≥ Grade 3 No. Patient (%)	Dose level (DL1/-1/-2)
Fatigue	17 (81 %)	5/7/5	6 (28 %)	2/1/3
Edema (limbs)	13 (62 %)	5/5/3	1 (5 %)	0/1/0
Anorexia	12 (57 %)	3/5/4	1 (5 %)	0/0/1
Lymphopenia	12 (57 %)	4/3/5	6 (28 %)	3/1/2
Nausea	11 (52 %)	5/2/4	0	−
Thrombocytopenia	10 (48 %)	4/2/4	1 (5 %)	0/0/1
Maculopapular rash	9 (43 %)	3/3/3	0	−
Mucositis	9 (43 %)	3/4/2	1 (5 %)	0/1/0
AST elevation	9 (43 %)	2/5/2	3 (14 %)	1/1/1
Hyperglycemia	8 (38 %)	3/1/4	0	−
Diarrhea	7 (33 %)	2/2/3	1 (5 %)	0/0/1^a^
Hypertension	7 (33 %)	1/4/2	0	−
ALT elevation	6 (28 %)	1/2/3	2 (9 %)	1/0/1
Hypertriglyceridemia	6 (28 %)	2/2/2	2 (9 %)^a^	1/0/1^a^
Pleural effusion	4 (19 %)	2/0/2	1 (5 %)	0/0/1
Pneumonitis	2 (9 %)	2/0/0	0	−
Sepsis	1 (5 %)	0/0/1	1 (5 %)	0/0/1^a^
Multi-organ failure	1 (5 %)	0/0/1	1 (5 %)	0/0/1^b^

*ALT* Alanine aminotransferase, *AST* Aspartate aminotransferase

^a^Grade 4

^b^Grade 5

### Adverse events possibly related to treatment

AEs by dose level are listed in Table 3. The most common non-hematologic AEs related to treatment reported in more than 30 % of patients were fatigue (81 %), edema of limbs (62 %), anorexia (57 %), nausea (52 %), rash (43 %), oral mucositis (43 %), increased aspartate aminotransferase (43 %), hyperglycemia (38 %) and diarrhea (33 %). Common hematologic AEs included anemia (43 %), lymphopenia (57 %) and thrombocytopenia (48 %). The most frequent G ≥ 3 AEs were fatigue (28 %) and lymphopenia (28 %). Five AEs of G ≥ 4 occurred: one patient in DL-2 developed G4 hypertriglyceridemia and there was a treatment-related death associated with G4 diarrhea, sepsis and G5 multi-organ failure (details described above). No dose-related trend in the incidence or severity AE was observed. During treatment, 7 patients required dose reduction (33 %) or had 1 of the 2 drugs discontinued due to AEs.

### Antitumor activity

**Fig. 1:**
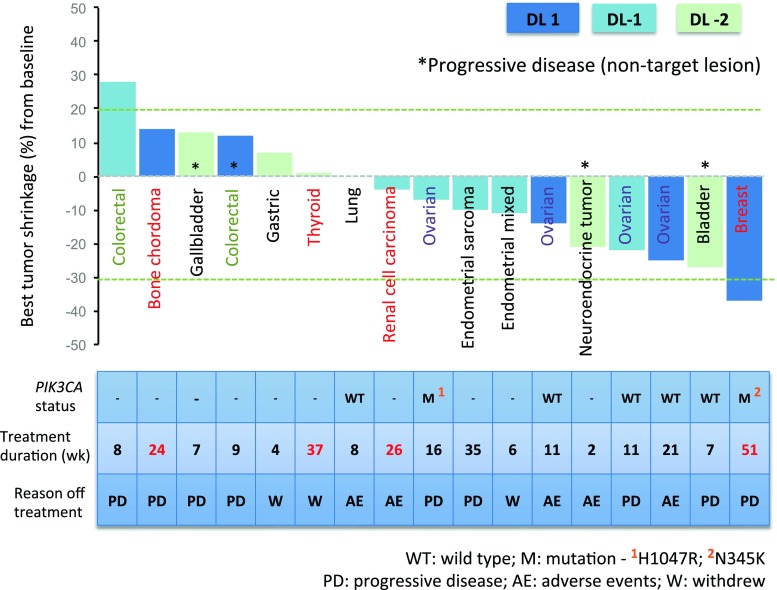
Waterfall plot of best treatment response

Eighteen of the 21 enrolled patients had at least one post-baseline tumor evaluation for treatment response. Figure 1 summarizes the antitumor response and duration of treatment in each evaluable patient. Six patients (33 %) had progressive disease (PD) as best response. One patient with inflammatory hormone receptor (HR)-positive HER2 negative breast cancer who had received 11 prior lines of treatment achieved partial response (PR) and remained on study for 51 weeks. Eleven patients (61 %) had stable disease (SD), in which 4 had prolonged SD for 24 weeks or longer. Tumor type, best response, and progression free survival (PFS) of last line of treatment prior to recruitment, as reference of growth modulation for these 4 patients, are as follows: 1 renal cell carcinoma (SD, 7 months), 1 thyroid cancer (PR, 7.5 months), 1 endometrial sarcoma (PD, 1 month), and 1 bone chordoma (PD, 1.5 month). This information suggested that the prolonged disease stabilization observed in the patients with endometrial sarcoma and bone chordoma was probably related to study drugs rather than slow growing biology.

### Correlative studies

#### PIK3CA mutation status

Tumor tissue for molecular profiling was available for 8 patients. Two cases with *PIK3CA* mutation were observed in which 1 patient had best response of PR (breast cancer, *PIK3CA* mutation at N345K, treatment duration 51 weeks) and another patient had SD (ovarian cancer, *PIK3CA* mutation at H1047R, treatment duration 16 weeks). Correlation of *PIK3CA* mutation with treatment response was not done due to small sample size.

#### Circulating angiogenic factors (CAFs)

**Fig. 2:**
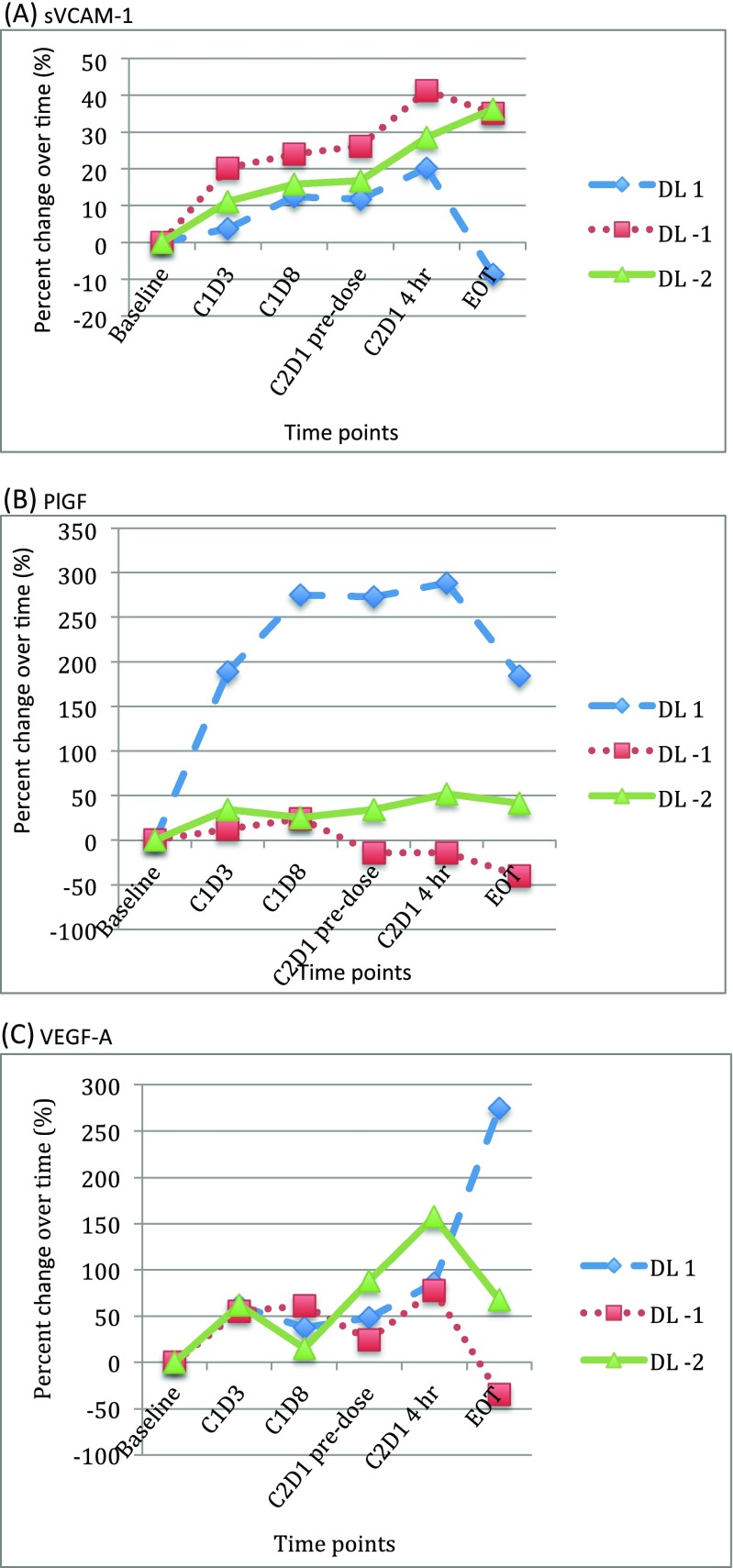
Average percent change of various circulating angiogenic factors during treatment. **a** sVCAM-1; **b** PlGF; **c** VEGF-A

There was a trend for sustained elevation of sVCAM-1 following treatment but it did not reach statistical significance. The magnitude of sVCAM-1 elevation did not appear to be related to the dose of trebananib or temsirolimus (Fig. 2a). For PlGF, there appeared to be elevation of this CAF with the highest dose of temsirolimus (DL1) (not statistically significant) that was not observed with the 2 lower dose levels (Fig. 2b). The change in VEGF-A over time did not show a specific trend and there was significant overlap in values between different dose levels (Fig. 2c). No correlation between the baseline values of CAFs and duration of treatment was detected (data not shown).

#### Tie2-expressing monocytes (TEMs) and thymidine phosphorylase (TP)

Blood for TEMs and TP analysis from the 11 patients treated in Princess Margaret Cancer Centre was available. No discrete population of Tie-2 expressing cells was present in peripheral blood. No association between either baseline TP or Tie2 M/L and radiographic response was observed, and no consistent pattern of change in TP or Tie2 M/L was observed during study therapy (Supplementary figure 3).

### Pharmacokinetics (PKs) and anti-trebananib antibodies

Sparse PK analysis of trebananib taken during cycle 2 is shown in Supplementary figure 4. There was more than 10-fold increase in plasma level of trebananib after 10 min of temsirolimus dosing at all dose levels (*p* < 0.01). The plasma concentration of trebananib returned to trough levels on D8. The post-dose plasma concentration trebananib for DL-1 (temsirolimus 20 mg, trebananib 15 mg/kg) was significantly higher than DL-2 (temsirolimus 20 mg, trebananib 10 mg/kg) (*p* < 0.01). Similar trend was observed for DL1 (temsirolimus 25 mg, trebananib 15 mg/kg) but did not reach significant difference, likely related to an exceptionally lower value for one sample. The post-dose and trough levels of trebananib in DL-2 were similar to the first-in-human phase I trial [12]. Similarly, the post-dose and trough levels of trebananib in DL1 and DL-1 with trebananib 15 mg/kg dosing were between the values at 10 mg/kg and 30 mg/kg observe in the phase I trial, suggesting that temsirolimus might not affect the PK of trebananib. No anti-trebananib antibodies were detected during treatment or at end of treatment.

## Discussion

This study explored the feasibility of combining the ANG1/2-Tie2 inhibitor trebananib with temsirolimus. The MTD was exceeded at the lowest dose level of trebananib 10 mg/kg weekly and temsirolimus 20 mg weekly. No tolerable dosing regimen could be identified for the combination. Frequent overlapping toxicities including fatigue, limb edema, anorexia, rash, mucositis, as well as lymphopenia, thrombocytopenia and transaminase elevation were seen. As a result, RP2D could not be identified, and the study was terminated. No further dose de-escalation is planned.

Sparse PK sampling of trebananib during cycle 2 showed similar drug levels as those observed from prior studies for the 10 mg/kg dose and PK simulations for 15 mg/kg [12]. Plasma concentrations of temsirolimus were not assessed in our study, making it difficult to exclude a drug-drug interaction between temsirolimus and trebananib as a cause for the toxicities observed. Although trebananib monotherapy has a favorable toxicity profile and the addition of trebananib to cytotoxic chemotherapy was well tolerated [13], combinations with other anti-angiogenic drugs have proven to be much more challenging. The AEs observed with the trebananib and bevacizumab combination included fatigue, diarrhea, nausea and epistaxis, whereas those of combining with motesanib were hypertension, diarrhea, fatigue and gastrointestinal upset [20]. Trebananib plus sorafenib led to diarrhea, palmar-plantar erythrodysesthesia syndrome, alopecia, hypertension and fatigue [21, 22]. The trebananib and sunitinib combination showed common AEs of diarrhea, fatigue, and hypertension [21]. The patterns of toxicities in our study were comparable to those of other anti-angiogenic combination partners tested with trebananib.

The toxicities that we observed were independent of dose level, and greatly exceeded the severity and frequency reported for each agent as monotherapy [23]. These toxicities mirror the experience of combining temsirolimus with other anti-angiogenic agents, suggesting the possibility of a pharmacodynamic interaction between temsirolimus and trebananib that leads to excessive toxicity. Temsirolimus in combination with bevacizumab in hepatocellular carcinoma demonstrated significant toxicities including cytopenia, fatigue, mucositis and diarrhea [24]. Likewise, this combination in advanced RCC was associated with a 43 % rate of treatment withdrawal unrelated to disease progression and 36 % incidence of G3 or G4 toxicities [25]. Temsirolimus has also been combined with oral tyrosine kinase inhibitors with anti-angiogenic effects. The combinations of temsirolimus with pazopanib were intolerable due to significant fatigue, leukopenia and electrolyte disturbances [26]. In the phase 1 study of temsirolimus with sunitinib, DLTs observed included rash, thrombocytopenia, stomatitis, diarrhea and stomatitis, and the combination was also found to be intolerable [27]. Based on these experiences, any further studies of temsirolimus with anti-angiogenic targeted drugs should be pursued with great caution.

There was one partial response in a breast cancer patient with *PIK3CA* mutation. Although the addition of the oral mTOR inhibitor everolimus to endocrine therapy improves progression-free survival in breast cancer [28], temsirolimus showed limited activity [29]. The exact mechanism for this patient’s exceptional response to the study drugs is unclear. Targeted mutational profiling was only performed on a subset of patients, limiting our ability to define predictive response biomarkers.

This study explored CAFs and their relationship with treatment. Although there appeared to be a trend of sVCAM-1 level elevation upon dosing, it was not statistically significant. Analysis of CAFs did not appear to be particularly informative. This highlights the challenges of interpretation of CAF data in anti-angiogenic trials, due to the lack of biomarker standardization, pathway redundancies, and variability of treatment administration and CAF assessment.

We investigated the role of circulating TEMs in predicting clinical efficacy. While measurement of Tie2 and TP in monocytes by flow cytometry was technically feasible, we did not identify discrete populations of Tie2+ and Tie2- monocytes, and instead used a relative measure of surface expression. Our exploratory analyses, which are limited by the low number of evaluable patients with TEMs data (only patients at Princess Margaret Cancer Centre had these assays performed because of requirement for rapid sample processing), found no association between these measurements and response to study treatment. Because mTOR inhibition may have direct effects on monocytes, it is possible that these assays could have greater utility in the setting of a Tie2/ANG directed monotherapy.

In conclusion, the combination of trebananib and temsirolimus was poorly tolerated. Toxicities did not appear to be dose-related suggesting there might be pharmacodynamic interaction between the two drugs. Other approaches to circumvent resistance to anti-angiogenic agents are needed, although potentiation of toxicities may limit the tolerability of anti-angiogenic drug combinations.

## Electronic supplementary material

Supplementary Figure 3 (PDF 287 kb)

Supplementary Figure 4 (PDF 40 kb)
