# Supplementary material for: A phase I trial of ANG1/2-Tie2 inhibitor trebaninib (AMG386) and temsirolimus in advanced solid tumors (PJC008/NCI♯9041)
Source: Invest New Drugs. 2015 Dec 19;34:104–11. doi: 10.1007/s10637-015-0313-8 (PMC4718956; doi:10.1007/s10637-015-0313-8)

Supplementary Figures

Figure 3. Analysis in changes in Tie2-expressing monocytes (TEMs) activities
(Blue lines denote patients with "target lesion tumor shrinkage" as best response)

(A) Tie2 M/L

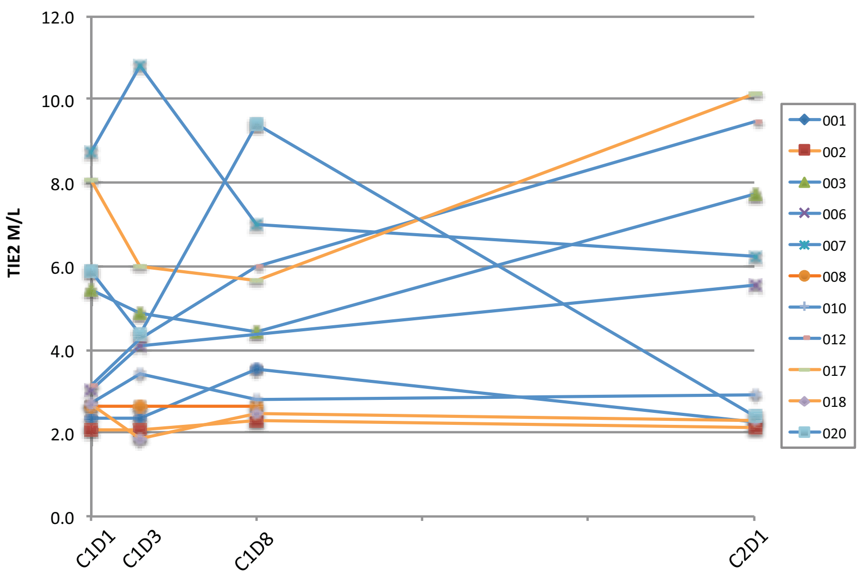

(B) TP M/L

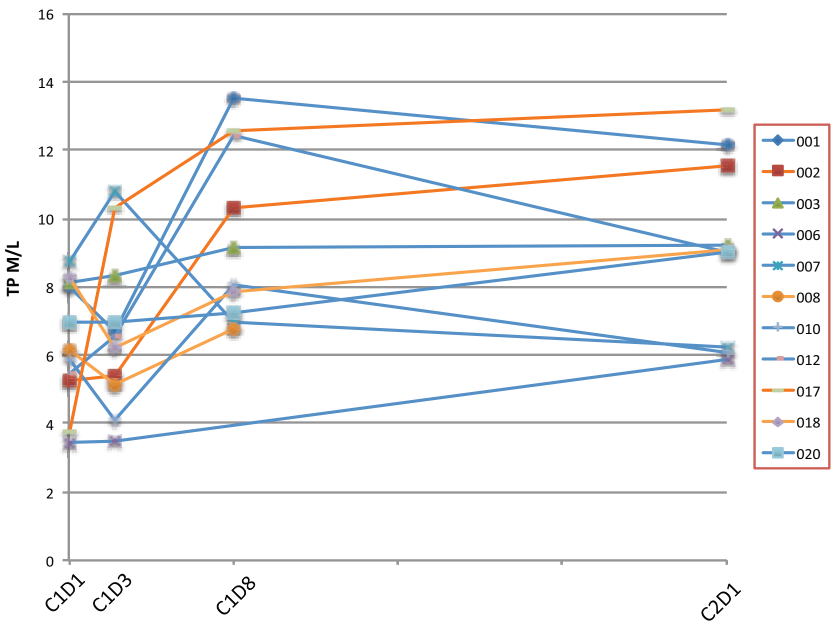

Supplement: Supplementary file 1 — (PDF 287 kb) [file 10637_2015_313_MOESM1_ESM.pdf]
