# Supplementary material for: A phase I trial of ANG1/2-Tie2 inhibitor trebaninib (AMG386) and temsirolimus in advanced solid tumors (PJC008/NCI♯9041)
Source: Invest New Drugs. 2015 Dec 19;34:104–11. doi: 10.1007/s10637-015-0313-8 (PMC4718956; doi:10.1007/s10637-015-0313-8)
Supplement: Supplementary file 2 — (PDF 40 kb) [file 10637_2015_313_MOESM2_ESM.pdf]

Figure 4. Sparse pharmacokinetic analysis of trebananib during cycle 2

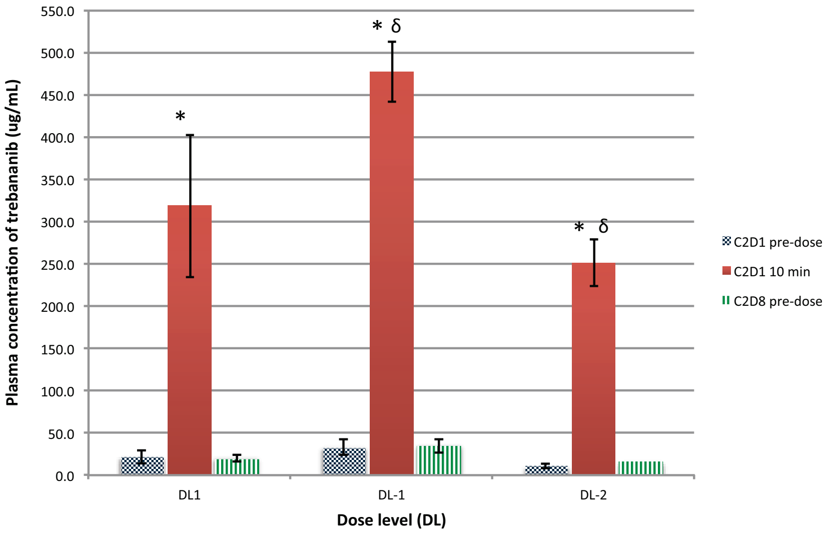

*p < 0.01 (compared with C2D1 pre-dose or C2D8 pre-dose for each dose level);
[δ]p < 0.01 (comparing C2D1 10 min plasma concentration between DL-1 and DL-2)
